# St. George's Hospital

**Published:** 1829-07

**Authors:** 


### HOSPITAL REPORTS,

(*Principally condensed from various Periodical Publications.*)

### ST. GEORGE'S HOSPITAL.

### *Case of lacerated Wound of the Thigh.*

George Jacobs, aged twelve, was admitted into the accident ward in this hospital, on Monday, May the 4th, with severe and extensive lacerated wound of the right thigh, occasioned by a heavy piece of timber having fallen on the limb, six or seven hours previously, at Twickenham. The laceration extended from half-way up the outside of the thigh to below the knee-joint; the fascia lata was extensively denuded, and in several places torn through, together with the vastus externus muscle. About the knee was some glairy fluid; but whether it came from a wound of the joint or merely from the supra-patellar bursa, could not be ascertained. The edges of the skin were separated transversely for two or three inches, but there appeared to be no loss of it. The pulse was weak, and the skin cold. The wound was dressed with lint and the many-tailed bandage; and a little wine given to rouse the patient.

Mr. Brodie, at his next visit, disapproving of this mode of dressing, applied three or four broad straps of adhesive plaster, without any intervening lint, so as to bring the edges of the skin in some degree into apposition. The limb was rolled with the common roller, and laid out, gently semiflexed at the knee, upon pillows.

On the 5th, he was ordered salines, with Sulphate of Magnesia at bedtime. Vini rubri Oss. ter die.

6th.—The pulse is frequent, the skin hot, and tongue furred. He was bled to ℥vi.; and, as his bowels had not been moved, he was ordered, Calomel gr. v. statim sumend.; Haust. Sennæ ℥i. post horas duas. Rep. Mist. vespere. Ol. Ricini ℥ss.

7th.—Hot and thirsty; tongue white, pulse quick; bowels have not acted. (The senna occasioned sickness.)—Rep. Ol. Ricini.

8th.—Much the same; hot, restless, and irritable, with anxious countenance and feverish look. There is a free discharge of fetid matter from the wound. The bowels have acted freely.

9th.—He is still in the same unfavorable condition as far as the constitutional affection is concerned, which partakes much more of the irritative than the inflammatory stamp, the skin being hot, tongue white and dry, pulse quick and irritable. Matter having collected at the under part of the thigh, a counter opening was made for its discharge in that situation.—℞ Haust. Salin. cum Vinæ Antim. tart. ♏x. sextis horis.

Next day, his bowels were again confined, and he required another dose of castor oil. Great irritation of the system; face remarkably fallen and anxious; the discharge copious, and extremely offensive to the smell.

11th.—The cellular membrane above the fascia of the leg, at its inner side, is in a sloughy state; as is also the fascia itself at the lower part of the thigh; added to this, a considerable quantity of matter has collected in the ham and around the knee-joint; the leg is swollen, skin discoloured, and a vesication has formed over the inside of the calf. Mr. Brodie made two or three very free incisions where the skin and subcutaneous textures appeared to have suffered most, and gave issue to a large quantity of fetid matter mixed with slough. The wounds were dressed with simple cerate spread on straps of linen; and he was ordered the following medicines: Haust. Salin. cum Liq. Opii Sed. ♏v. sextis horis.

12th.—He appears to be in much danger. The pulse is rapid and weak; tongue dry, but not brown; face sunken, with a hectic flush upon the cheeks; occasional rambling; great depression. The discharge very copious; sloughing does not appear to be extending. The symptoms, indeed, are rather of a hectic than typhoid character.—Rep. Haust. Salin. cum Liq. Opii Sed.

13th.—Something better today; skin cooler, tongue cleaner, pulse less frequent; discharge from the wound becoming healthy, though very abundant.

On the next day he was little altered, save that the depression seemed gradually increasing.

15th.—Has vomited two or three times; pulse very quick, small and feeble; bowels open; discharge from the leg very great. In several places the fascia and cellular membrane form sloughs, that have not yet come away, intermingled with pus and dirty-looking granulations.—Ordered to leave off all his medicines, and to take beef-tea; Liq. Opii Sed. ♏xx. at bedtime; Vini rubri Oss. Porter Oj. quotidie.

16th.—Evidently sinking fast: pulse rapid and sharp; tongue brown; face hippocratic; mind wandering; the toes are bluish and cold. Vomited again in the night, but no rigor could be traced. Belly rather tense, and painful upon pressure.

He died at seven A.M. of the 17th, vomiting a quantity of coffee-ground matter in the very act of dying. The nurse distinctly states he had no rigor.

*Post-mortem Examination.*—The thoracic and abdominal viscera were sound. The foot was mottled in appearance, with small vesications about the ankles. The knee-joint did not appear to be opened into, but, on exposing it, the inner surface was found in much the same sloughy-looking state as the external parts. The capsule at the upper part seemed to be destroyed, and the joint to communicate with a sloughy abscess in the centre of the crureus muscle. The cartilages were not ulcerated. The fibula was broken, about the junction of the middle with the upper third, and its' head was quite dislocated backwards from the tibia. There was still another fracture of the fibula lower down. The head of the bone must have been exposed in the sloughy abscess of the soft parts, which was very extensive, reaching from more than half-way up the thigh to the same distance down the leg, and in various directions. The muscles appeared to be sound immediately beyond the disorganized parts.

---

### *Compound Fracture of the Leg.*

WILLIAM NORRIS, aged seventy-five, admitted into St. George's Hospital, May 1st, under the care of Mr. BRODIE. Tibia fractured obliquely a little below the middle of the leg; fibula fractured lower down; two small wounds of the integuments on the inner side, communicating with the fracture of the tibia; not much extravasation around. The injury was inflicted by the wheel of a coach passing over the limb. The patient is a hale-looking old man.—Leg placed in junk. Lot. spirit.

On the 2d, the fracture-box was substituted for the junk. For the first four or five days the leg was easy, and nothing particular occurred.

6th.—As his bowels were a little confined, he was ordered

Haustus Sennæ. In the course of the day he was observed to be incoherent, and in the night was so delirious as to require confinement. He took Tr. Opii gtt. xv. and twenty drops more in the course of an hour.

Next morning he was quieter; pulse weak, skin cool, tongue clean; no headach. Being rather low, he was ordered Vini rubri ℥iv.; Haust. Cinchonæ ℥i.; Confec. Aromaticæ ℈ss. ter die.

On the 8th, the pulse was stronger, but intermittent; no delirium. Slept without opium.

9th.—A dark-coloured erysipelatous blush, which appears to implicate the cellular texture as well as the skin, occupies the leg from the ankle to the knee.

11th.—The cellular membrane was now in a sloughy condition, for a considerable distance up and down the leg, especially on its inner side; the skin was of a dark, dusky, red tint; foot warm; no gangrene, but the cuticle about the centre of the tibia separated by vesication from the cutis. He rambles at times; pulse weak, tongue clean. Took table-beer, wine, middle diet, and Tr. Opii gtt. xv. h. s. Cold lotion was applied to the part. Incisions were made at various times in the leg down to the cellular membrane, and sloughs of that, mixed with foul purulent matter, evacuated.

The state of the limb was little ameliorated. The erysipelatous blush extended up the thigh on either side for three or four inches above the knee, but the sloughing of the cellular tissue appeared to be confined exclusively to the leg. The foot and limb always retained their natural temperature. During this local affection of the parts, the pulse was rapid and weak, the mind wandering, but at no period since the 6th was there actual delirium; tongue dry and brown; features sunken. He was ordered Haust. Cinchonæ ℥iss.; Confec. Aromatica gr. xv.; Tinct. Opii ♏v.; Amm. Carb. gr. v. ter die. Porter Oj.

13th.—Vini rubri Oss. quotidie. Amm. Carb. gr. v.; Confec. Arom. gr. xv.; Liq. Opii Sed. ♏x.; Aqua ℥iss. ft. haust. sex. hor.

On the 14th, Mr. Brodie made some more incisions, giving issue to foul pus, and exposing the rotten subcutaneous textures. The patient was evidently sinking. Ordered an ounce of brandy in addition to the other stimuli.

On the 17th, he was plainly in *articulo mortis.*

18th.—Died early this morning.

*Sectio cadaveris.*—The limb was not in a state of gangrene, but the greater portion of the anterior aspect of the leg, for more than its middle third, was sloughy. The tibia, throughout the greater part of this extent, was exposed, but not denuded of its periosteum, which was vascular and inflamed. The muscles below the sloughy part were sound, but remarkably congested with black blood, as if that were a state preceding the actual sloughing. The mischief was mainly confined to the superficial cellular texture. The fracture of the tibia was oblique, the lower portion was comminuted, but the fracture apparently did not pass into the ankle-joint. The foot was a good deal tumefied, the skin discoloured, and the cuticle separated in one or two places by partial vesication.

The thoracic viscera healthy, and also the abdominal, with the exception of the liver, which showed symptoms of the dram-drinking degeneration. The aorta was dilated at its arch, especially at the convex part of it. About the dilated portion were two large patches of atheromatous deposition, apparently between the outer and middle coat.

---

### *Remarkable Instance of the Constitutional Nature of Fungus Hæmatodes.*

On the 24th of last April, the dissection of Mary M'Carthy, ætatis thirty-two, took place in St. George's Hospital.

*Head.*—On turning down the flaps of the scalp, there appeared upon the calvarium several tumors, looking like those of fungus hæmatodes, and all receiving a covering of sound and unbroken, but thin, pericranium. They were three in number. One was situated on the frontal bone, over the superciliary ridge, towards its outer angle: its circumference equalled that of a small orange, and its greatest projection was nearly an inch above the level of the neighbouring bone. The second tumor was seated over the left parietal bone, at the top of the head, and not so large as a half-crown piece. The third was nearly, if not quite, the size of the first, placed far back in the occipital region, to the right of the median line.

The calvarium was removed with the dura mater attached to it. The membrane, looking from within, was found to be free from ulceration at the site of the tumors, but under each it presented an injected and inflamed appearance, more or less determinate. The dura mater was stripped from the bone, when it was found to be attached to the root of each tumor by adhesions, which, however, were readily torn through.

The tumors themselves were of the genuine medullary character. They were in contact with the pericranium externally, with the dura mater internally, and the intermediate bone was more or less absorbed, and blended in spiculæ with the diseased mass. The degeneration had demonstrably commenced in the diploë, for the destruction of this was much more extensive than that of either of the outer tables: indeed, the medullary matter was in some parts contained in the diploë, whilst the outer and inner tables yet remained entire. The tumor at the corner of the eye had made its way through into the orbit, and had not pierced the periosteum of that cavity. Nothing unusual was observed about the brain, or its more immediate investing membranes.

*Thorax.*—There was no effusion into the cavity of the pleuræ, nor other marks of inflammation of the membrane. The lungs presented in one or two parts small medullary deposits.

*Abdomen.*—The liver presented an exquisite specimen of the disease. Medullary tumors, of various sizes, were found in its substance, and towards the under part of the left lobe was a very large one. In those which were most mature, a kind of ulcerated cavity was contained in the centre. In all, the outer portion constituted a sort of cortical substance, finer grained and more compact than the central, which was coarse and rough. In each of the kidneys were several cysts, containing a drachm or more of greenish fluid, with membranous septa running across them. They were chiefly in the tubular substance of the viscus. In several parts of the cortical substance were pale, fibrous-looking patches, not tumors, resembling what are frequently met with in the organ. There was much thickening about the cervix uteri, and the section showed a surface really not unlike commencing medullary fungus.

In the situation of the left mamma was a tumor the size of a walnut, of medullary structure; and towards the axilla were one or two more, apparently lymphatic glands, disorganized in this way. The right mamma, which was shrunken and withered, had not, when cut into, a natural appearance.

Over the left patella was a diseased bursa. It was a perfect cyst, and readily dissected from the subjacent ligament and bone. Its walls were quite cartilaginous, and three or four lines in thickness. Its cavity contained a yellowish lacerable substance, half lymph, half pus.

So much for the dissection, which we have given first, on account of its very interesting character; and we shall now proceed to relate briefly the history of the case.

The patient was first admitted into the hospital on the 24th of December, 1828, with a malignant-looking, ulcerated tumor of the left breast, of seven months' duration, and originating, it would seem, in suppression of the milk. It had first ulcerated three weeks prior to admission, and hemorrhage had occurred more than once during that short period. The fungus being in a foul and very sloughy state, and resembling carbuncle in no slight degree, Mr. Keate made a crucial incision into its substance. The bleeding which ensued was arrested by pressure with lint and a roller; but hemorrhage took place every now and then, the surface became no cleaner, and on the 1st of January the whole of the breast was removed by amputation. An enlarged gland in the axilla, which the patient stated to have been there before the disease in the breast commenced, we rather think was left. The section of the amputated tumor of the breast showed it to consist of the medullary sarcoma, run into ulceration on its surface.

The operation was followed by no very urgent or alarming symptoms, though nothing like healthy union or a speedy convalescence ensued. She continued to be harassed from time to time by attacks of vomiting, with much irritation and depression of the system; and on the 6th the parts around the wound were invaded by erysipelas. This attack was not severe, and speedily gave way to the bark and supporting system. On the 16th, a large abscess was discovered to be forming in the right nates; and on the 26th it was opened, and a considerable quantity of pus discharged. By the 7th of February, the wound on the chest was nearly healed; and she was made an outpatient on the 18th, at her own particular request.

Previously to this she had complained of pain about the shoulders, and thickening and induration were but too apparent at the axillary margin of the pectoral muscle on the amputated side. Need we say that the prognosis was gloomy in the extreme.

On the 1st of April she re-applied for admission, in a melancholy condition. In the interval between the date of her leaving the house and that of her re-entering it, tumors, apparently malignant, had formed upon three different parts of the head. She complained of constant pain in that region, and the right side of the face was partially paralysed. In the axilla, or its margins, several indurated tumors were felt, and one or two in the cicatrix of the former wound.

It would be useless to particularize the treatment had recourse to, as not the slightest benefit ensued from its employment. She gradually sank into an apathetic state, with the mouth distorted, and more or less paralysis of many other parts. The tumors grew, but did not ulcerate; and in this miserable condition death at length put a period to her deplorable existence.

The rapid progress of the tumors on the head is a very remarkable circumstance, and, taken in connexion with the presence of the disease in the lungs and liver, serves to place in, unhappily, too broad a light the constitutional seat of medullary sarcoma. The case will also serve to show how small the chance of success from amputation is, when performed after the tumor has gone into ulceration.*

